# A medical student scholarly concentrations program: scholarly self-efficacy and impact on future research activities

**DOI:** 10.1080/10872981.2020.1786210

**Published:** 2020-06-26

**Authors:** Rebecca M. DiBiase, Mary Catherine Beach, Joseph A. Carrese, Jennifer A. Haythornthwaite, Sarah J. Wheelan, Meredith A. Atkinson, Gail Geller, Kelly A. Gebo, Jeremy A. Greene, Stephen M. Sozio

**Affiliations:** a Department of Medicine, McGaw Medical Center of Northwestern University, Chicago, IL, USA; b Department of Medicine, Johns Hopkins University School of Medicine, Baltimore, MD, USA; c Welch Center for Prevention, Epidemiology, and Clinical Research, Johns Hopkins Medical Institutions, Baltimore, MD, USA; d Berman Institute of Bioethics, Johns Hopkins University, Baltimore, MD, USA; e Department of Health, Johns Hopkins Bloomberg School of Public Health, Baltimore, MD, USA; f Department of Psychiatry & Behavioral Sciences, Johns Hopkins University School of Medicine, Baltimore, MD, USA; g Department of Oncology, Johns Hopkins University School of Medicine, Baltimore, MD, USA; h Department of Molecular Biology and Genetics, Johns Hopkins University School of Medicine, Baltimore, MD, USA; i Department of Pediatrics, Johns Hopkins University School of Medicine, Baltimore, MD, USA; j Department of History of Medicine, Johns Hopkins University School of Medicine, Baltimore, MD, USA; k Department of Epidemiology, Johns Hopkins Bloomberg School of Public Health, Baltimore, MD, USA

**Keywords:** Self-efficacy, scholarship, scholarly concentrations program, undergraduate medical education

## Abstract

**Background:**

The Scholarly Concentrations program was established at Johns Hopkins University School of Medicine in 2009 with the aim of instilling passion for scholarship.

**Objective:**

Our study aimed to determine whether the Scholarly Concentrations program achieves positive changes in medical student self-efficacy in conducting research and, if so, whether this results in future career aspirations toward scholarship.

**Design:**

We used the Clinical Research Appraisal Inventory-Short Form (CRAI-SF) to assess changes in self-efficacy among students completing the Scholarly Concentrations program between 2014 and 2017. We calculated composite mean scores of six domains. We included outcomes on whether students published a manuscript, overall program perceptions, and likelihood of future research careers. We analyzed relationships between CRAI-SF scores and outcomes using paired t-tests and multivariable-adjusted logistic regression.

**Results:**

A total of 419 students completed the Scholarly Concentrations program. All 6 CRAI domain scores showed significant improvements in self-efficacy between the pre-Scholarly Concentrations and post-Scholarly Concentrations ratings (range of changes 0.76–1.39, p < 0.05 for all). We found significant associations between post-Scholarly Concentrations self-efficacy ratings and course satisfaction (adjusted OR 1.57 [95% CI 1.20, 2.07]) and mentor satisfaction (OR 1.46 [1.15, 1.86]), as well as students’ intent to conduct future research (OR 1.46 [1.15, 1.86]). These results were robust to sensitivity analyses, and pronounced in the group of students without prior research experience.

**Conclusions:**

Our findings suggest that a Scholarly Concentrations program is associated with an increased self-efficacy for research, and these changes in self-efficacy are associated with higher satisfaction in the scholarly experience and increased likelihood of pursuing scholarly work. Other medical schools could use such a tool of self-efficacy to both investigate the overall Scholarly Concentrations experience and understand factors that may increase interest in future physician-scientist pathways.

## Introduction

Career choice for medical students, and whether this includes an academic career, continues to be high a priority area for research and advising [1,2]. Bandura’s Social Cognitive Theory on self-efficacy lays some of the groundwork for the determinants of career choice [3], which is useful as we consider factors influencing our rising physicians. Perceived self-efficacy is a mechanism of personal agency largely influenced by one’s judgments of their capabilities [4,5]. It may motivate students making pivotal career decisions based on where they believe they will be most fulfilled and successful. Self-efficacy is a dynamic appraisal that interacts with contextual factors such as values, aptitude, and goals [6] and programs can be built to increase self-efficacy and assess these changes. Considering the prominent role of research interest in forming academic career selection [7], it follows that educational interventions have the potential to modify self-efficacy in conducting research and help shape students’ career decisions.

The development of research interest through Scholarly Concentrations (SC) programs has increased across U.S. medical schools. These programs are based on the premise that human agency is essential in career development, and fostering self-efficacy and passion for research will help expand academic medicine [8,10]. Providing mentorship and the flexibility to investigate medical areas of uncertainty are two ways to instill this passion for an area of scholarship, and can be utilized to build a Scholarly Concentrations curriculum personalized to students [11,12]. A meta-analysis of papers evaluating various SC programs across the country suggested that students were willing to undertake SC projects again if provided the opportunity, and that most were satisfied with their mentor [13]. However, the authors of this same meta-analysis suggested the need for future studies to identify areas of programmatic success, evaluate student performance using standardized criteria, and address the question of ‘why programs work.’

Johns Hopkins School of Medicine first established their SC program in 2009. In this paper, we review longitudinal data exploring the changes in student self-efficacy for scholarship that occur during the SC program, and whether these changes then translate into inspiring students both in their course experience and in their pursuit of future scholarly activities.

## Materials & methods

### Study population

The SC program has been described previously [14,15]. Briefly, at the beginning of the SC program, students choose from one of five concentrations to pursue (Basic Science, Clinical Research, History of Medicine, Humanism & Ethics in Medicine, and Public Health). Students meet their SC advisors to help clarify their interests and then review potential mentors from lists of Hopkins departmental and research faculty and prior Hopkins SC mentors. Overall, the SC program occupies 55.5 curricular hours spanning 18 months. Each educational module is structured in a one-week period, with 9 hours total per week. Four modules are taught in the first year and two in the second. Most students conduct a significant portion of research in the summer between the first and second years of medical school.

For our study population, we included all students at the Johns Hopkins School of Medicine who completed the Scholarly Concentrations program. Although the program began in 2009, our first cohorts using the survey instruments described below completed the course between 2014 and 2017. We included data on these cohorts and follow-up data on these cohorts through medical school year 5 (if additional time taken) or post-graduate year 1. Because participation in the SC program is a required component of the preclinical M.D. curriculum at Johns Hopkins, all eligible study participants were enrolled in their first and second years of the M.D. program. M.D.–Ph.D. program students are excluded from the Scholarly Concentrations program and this analysis.

### Self-efficacy score

The Clinical Research Appraisal Inventory (CRAI) was developed as a tool to measure self-efficacy for conducting research among physician-scientists in training [16]. It contains 46 items in a 10-point Likert-type response format, and requires approximately 15 minutes to complete. The CRAI-Short Form (CRAI-SF) is an abridged version [17]. We administered the CRAI-SF to our students via the Qualtrics online survey platform prior to the beginning of the SC program and directly after its completion. To ensure the questions on the CRAI-SF were appropriate to the concentration assessed, we omitted items not relevant for that concentration. This resulted in a total of 4/46 removed for Basic Science, 12/46 removed for History of Medicine, and 1/46 modified for Humanism & Ethics in Medicine. Even after doing so, each domain was still represented by more than three questions (See Appendix section Supplementary Methods: Descriptions of Specific Variables Comprising Mean CRAI Domain Scores). We used responses to both the pre-and post-program surveys to calculate composite scores for the following six domains: 1) conceptualizing a study; 2) organizing a study; 3) protecting research subjects and responsible conduct of research; 4) collaborating with others; 5) study design and data analysis; and 6) reporting a study. We averaged the answers for the questions within each domain to derive the composite score for each domain, and then averaged these further to derive an overall mean CRAI score across domains. We listed questions contributing to each domain in the Supplement.

### Other important student factors in scholarship

We also investigated other student factors that may influence self-efficacy and/or interest in the future scholarship. We selected these variables *a priori* based on the factors we determined may be most important in impacting medical students’ experiences with scholarly work. The final choice of scholarly concentration category was one such variable. Options for this included Basic Science, Clinical Research, History of Medicine, Humanism & Ethics in Medicine, and Public Health. Another variable selected *a priori* included whether students had published scholarly work prior to starting the SC program; further descriptions of publication abstraction are provided below. Finally, we included students’ average scores on all CRAI domains prior to starting the SC program.

### Inspiration in the course and future activities

We included five primary short- and long-term outcomes reflecting Kirpatrick’s trajectory from satisfaction to behavioral change [18]. The first two involved self-reported student satisfaction ratings. The first outcome variable assessed students’ overall satisfaction with the SC course. Data were collected using the survey question ‘How would you rate the overall quality of this course?’ Response options were on a 5-point Likert scale: ‘Poor’, ‘Fair’, ‘Satisfactory’, ‘Very Good’, and ‘Excellent.’ Results were then dichotomized, comparing the highest satisfaction rating (‘Excellent’) to all other ratings. We used a very similar method to generate the outcome variable that represented students’ satisfaction with their research mentors. Students responded to a survey question ‘Overall, how satisfied were you with your mentor?’ as ‘Very dissatisfied’, ‘Dissatisfied’, ‘Neutral’, ‘Satisfied’, or ‘Very Satisfied.’ Results were again transformed into a dichotomous outcome variable, comparing ‘Very Satisfied’ with any other response.

Two additional outcome variables included whether students were authors on any published scholarly work after completion of the SC program, and whether they specifically were the first author on a manuscript. We collected the data for these variables by abstraction from the search engines, PubMed, Scopus, and Google Scholar, using students’ names and cross-referencing with ‘Johns Hopkins’, prior undergraduate institution, or subsequent residency institution. We made no distinction between different types of publications, such as case reports versus hypothesis-driven research. These publications were abstracted by the course administrator (Carly Wasserman, MAT), with weekly review for clarification and discussion with the senior author (SS). Due to the granularity needed to search for prior and future publications, we limited this search to those students who already provided this information in graduation materials, Doximity, LinkedIn, and other publicly available sources (i.e., only those students who finished the SC course from 2014 to 2016). For each student, we recorded the numbers of publications and numbers of first author publications as continuous variables. We then transformed these into dichotomous variables, comparing at least one publication (or at least one first-author publication) with no publications (or no first-author publications).

Finally, to understand whether students were interested in the future research, we asked in the post-SC course survey, ‘How did your experience with your scholarly project impact your plans for future research or scholarly work?’ Students responded with ‘More likely’, ‘Less likely’, ‘Same’, and ‘Unsure’. We compared those responding ‘More likely’ to all other responses.

We used prior literature to help determine that these variables would adequately capture both short-term and long-term validity measures in assessing satisfaction with the program and long-term interest in scholarship [19].

### Analytic strategy

We used paired t-tests to compare the difference between composite pre- and post-SC program mean scores from the CRAI-SF, with t-tests and ANOVA to compare the baseline variables’ associations with change in each CRAI-SF domains score. We used multivariable logistic regression to assess whether there was an association between mean domain scores after SC program completion and students’ perceived satisfaction and future research success and aspirations, as captured by the five outcome variables. We used a stepwise model-building approach, with unadjusted model results first examined, then a minimally adjusted model, and finally a fully adjusted model. The minimally adjusted model included students’ SC area. The fully adjusted model added publications prior to SC and ‘Pre-SC’ average CRAI score.

We used stratified logistic regression models to investigate whether self-reported interest in research modified the impact of final self-efficacy on outcomes. Results were stratified based on self-reported interest in research using the question: ‘When you started medical school, did you plan to conduct research or other scholarly work?’ Students chose between the responses: ‘Yes, definitely’, ‘Yes, probably’, ‘Unsure’, and ‘No’. We then aggregated the data into a dichotomous covariate, with the ‘Yes, definitely’ compared to any other response. We then included an interaction term in the fully adjusted model to examine whether any effect measure modification existed between the mean post-SC program CRAI domain scores and this dichotomous variable.

### Sensitivity analyses

In sensitivity analyses, instead of capturing the exposure variables as mean CRAI-SF Post-SC program scores on a continuous scale, we dichotomized this exposure variable to those who achieved the three highest domain scores (8–10 out of a potential 10 maximum), versus those who achieved any other score, to examine ceiling effects. We utilized a change variable capturing the difference between ‘Post-SC’ and ‘Pre-SC’ mean domain scores as the exposure instead of using the ‘Post-SC’ score as the primary exposure. We also examined the course satisfaction, mentor satisfaction, and publications during SC variables in their original continuous forms rather than categorical forms to determine whether the same patterns of significance were retained.

We performed analyses using Stata 15.0 (College Station, TX). A p-value <0.05 was considered significant. The Johns Hopkins Institutional Review Board approved this investigation (IRB00103412).

## Results

### Cohort characteristics

Four hundred nineteen students completed the SC program between 2014 and 2017. Baseline characteristics of the study population are summarized in Table 1. A much larger number of students chose the Clinical Research scholarly concentration (49%) than any other concentration subject. Most students (86%) reported they were either ‘definitely’ or ‘probably’ interested in conducting future research before starting the SC program curriculum, and the majority of students entered the program with prior publications (66%).

**Table 1.** Baseline Characteristics and CRAI scores of students in the Johns Hopkins SC program, 2014–2017.

Characteristics*	N (%) or Mean (SD)
**Year Completed SC Program** (N = 419)	
2014	102 (24)
2015	103 (25)
2016	108 (26)
2017	106 (25)
**Concentration** (N = 419)	
Basic Science	43 (10)
Clinical Research	203 (49)
History of Medicine	29 (7)
Humanism & Ethics in Medicine	36 (9)
Public Health	108 (26)
**Baseline Interest in Future Research** (N = 357)	
Yes, definitely	215 (60)
Yes, probably	93 (26)
Unsure	39 (11)
No	10 (3)
**Publications Prior to SC** (N = 313)	
Yes	207 (66)
No	106 (34)
**Baseline CRAI Domain Scores**	
Study Design & Data Analysis (N = 323)	5.5 (1.7)
Conceptualizing a Study (N = 323)	6.4 (1.7)
Collaborating with Others (N = 322)	6.4 (1.9)
Organizing a Study (N = 322)	6.5 (1.9)
Protecting Subjects & Responsible Conduct (N = 321)	4.8 (2.2)
Reporting a Study (N = 322)	5.9 (1.7)

Abbreviations: N: Study population size, SD: Standard Deviation, SC: Scholarly Concentrations, CRAI: Clinical Research Appraisal Inventory

*Baseline interest and baseline CRAI captured by student surveys. Publications abstracted only for those students who had the opportunity to graduate medical school (minimum two years after SC program completion).

**Figure 1.**
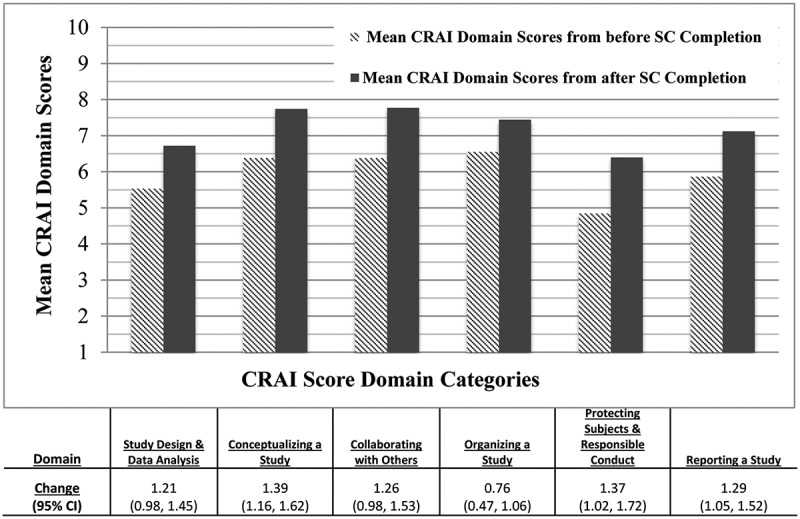
Relationship Between Pre- and Post-SC Program Completion Composite CRAI-SF Self-Efficacy Domain Scores.

Three hundred twenty-one students completed both the pre- and post-course CRAI-SF survey, a 77% response rate. On the CRAI-SF taken before starting the SC program, the lowest mean domain score (4.8) was in the ‘Protecting Subjects & Responsible Conduct’ domain, while the highest score (6.5) was in the ‘Organizing a Study’ domain. We depict comparisons between CRAI domain scores ‘Pre-SC program’ and ‘Post-SC program’ in Figure 1. We found a statistically significant positive change between the ‘Pre’ and ‘Post’ scores, with mean student scores improving in every domain. We observed the smallest change in the ‘Organizing a Study’ domain (change 0.76; 95% Confidence Interval (CI) [0.47, 1.06]), and the greatest change in the ‘Conceptualizing a Study’ domain (change 1.39, 95% CI [1.16, 1.62]). When examined by baseline characteristics, the only variables with a statistically significant association with the change in self-efficacy ratings were student concentrations (with ‘Conceptualizing a Study’ and ‘Collaborating with Others’) and baseline interest in research before the SC program (with ‘Reporting a Study’) [data not shown].

*Change calculated as the difference between post-SC and pre-SC CRAI-SF composite scores in each domain. Abbreviations: CRAI = Clinical Research Appraisal Inventory, using mean domain scores on the Post-SC questionnaire as measures of self-efficacy attainment; Regression Coefficient = Beta coefficient generated with linear regression models; 95% CI = 95% Confidence Intervals.

### Outcomes

**Figure 2.**
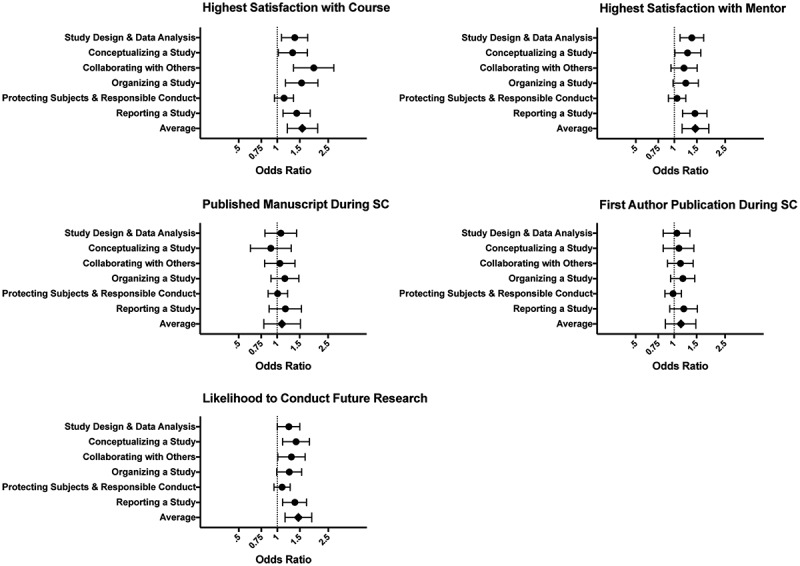
Associations of Average Self-Efficacy Score at End of SC Course with Outcomes, Across Domains and Overall Average.

We show unadjusted, minimally adjusted, and fully adjusted analyses of the post-SC average CRAI score across domains on the outcomes in Table 2. In addition, we depict results from the fully adjusted model (both by domain and overall average) in a forest plot form in Figure 2. We show results for each separate domain score in Supplementary Table 1.

**Table 2.** Associations of average self-efficacy score at the end of SC course with outcomes, in sequential models.

	Model 1		Model 2		Model 3	
Outcome	Odds Ratio	95% CI	Odds Ratio	95% CI	Odds Ratio	95% CI
Highest Satisfaction with Course	**1.56**	**1.24, 1.97**	**1.59**	**1.26, 2.01**	**1.57**	**1.20, 2.07**
Highest Satisfaction with Mentor	**1.42**	**1.17, 1.72**	**1.45**	**1.19, 1.78**	**1.46**	**1.15, 1.86**
Published Manuscript During SC	1.18	0.93, 1.51	1.16	0.90, 1.50	1.09	0.79, 1.52
First Author Publication During SC	**1.30**	**1.04, 1.62**	**1.26**	**1.01, 1.58**	1.13	0.85, 1.48
Likelihood to Conduct Future Research	**1.24**	**1.03, 1.50**	**1.24**	**1.03, 1.50**	**1.46**	**1.15, 1.86**

Abbreviations: CRAI = Clinical Research Appraisal Inventory, using the Post-SC questionnaire as measures of self-efficacy attainment; 95% CI = 95% Confidence Intervals.

Average CRAI Score obtained by averaging the scores on questions in each domain of the CRAI, and then averaging across the six domains.

Model 1: Unadjusted Model, containing only exposure and outcome variables.

Model 2: Minimally Adjusted Model, containing exposure and outcome variables, as well as SC concentration category.

Model 3: Fully Adjusted Model, containing exposure and outcome variables, covariates from Model 2, as well as publications prior to SC and average CRAI score before starting the SC curriculum.

Average CRAI Score obtained by averaging the scores on questions in each domain of the CRAI, and then averaging across the six domains. Results presented include the Fully Adjusted Model, containing exposure and outcome variables, choice of SC concentration, as well as publications prior to SC and average CRAI score before starting the SC curriculum.

In terms of the satisfaction variables, adjusted regression models demonstrated that the higher the achieved self-efficacy score, the more likely students were to report satisfaction with the course (OR 1.57, 95% CI [1.20, 2.07]) and their mentors (OR 1.46, [1.15, 1.86]). However, self-efficacy achievement was not found to be associated with either of the publication measures in final models (published during SC: OR 1.09, [0.79, 1.52]); the first author published during SC: OR 1.13, [0.85, 1.48]). Though the unadjusted and minimally adjusted models generated some significant results for the ‘First Author Publications during SC’ outcomes, results became less significant as additional covariates were included in the models, and no significant association remained with the publication outcome variables in the fully adjusted model.

A higher average post-SC CRAI score was also significantly associated with student plans to conduct future research in the fully adjusted model (OR 1.46, [1.15, 1.86]). In Supplementary Table 1, we observe that the specific domains in which this significant association was seen were ‘Reporting a Study’, ‘Organizing a Study’, and ‘Collaborating with Others.’ This was similar to the domains found to be significantly associated with ‘Satisfaction with Mentor.’

In terms of potential effect modification, we found the highest baseline level of interest to be significantly associated with higher course satisfaction ratings (OR 1.43, [1.02, 2.02]), while other baseline interest levels were not (Table 3). We did not find a statistically significant interaction in these results. In contrast, lower baseline interest levels were significantly associated with students’ reported satisfaction with their mentors (OR 1.60, [1.07, 2.41]) and their plans to conduct future research (OR 1.61, [1.04, 2.49]). In both of these relationships, we did not find statistically significant interactions.

**Table 3.** Associations of average CRAI score at the end of SC course with outcomes, stratified by self-reported baseline research interest.

	Highest Baseline Level of Interest (n = 378)		Other than Highest Baseline Level of Interest (n = 278)		
Outcome Variable	Odds Ratio	95% Confidence Interval	Odds Ratio	95% Confidence Interval	P-Interaction
Highest Satisfaction with Course	**1.43**	**1.02, 2.02**	1.63	0.99, 2.69	0.21
Highest Satisfaction with Mentor	1.38	1.00, 1.91	**1.60**	**1.07, 2.41**	0.90
Published Manuscript During SC	1.38	0.81, 2.37	0.78	0.46, 1.32	0.14
First Author Publication During SC	1.49	0.99, 2.23	0.71	0.43, 1.16	0.15
Likelihood to Conduct Future Research	1.31	0.98, 1.76	**1.61**	**1.04, 2.49**	0.33

Abbreviations: CRAI = Clinical Research Appraisal Inventory, using mean domain scores on the Post-SC questionnaire as measures of self-efficacy attainment; N = Study population size; P-interaction = P-value for the interaction term (significance level of p < 0.01).

Average CRAI Score obtained by averaging the scores on questions in each domain of the CRAI, and then averaging across the 6 domains. Results presented include the Fully Adjusted Model, containing exposure and outcome variables, choice of SC concentration, as well as publications prior to SC and average CRAI score before starting the SC curriculum.

An example statement to interpret Odds Ratio: ‘We found the highest baseline level of interest to be significantly associated with higher course satisfaction ratings (OR 1.43, [1.02, 2.02]), while other baseline interest levels were not.’ [also included in text]

### Sensitivity analyses

When we dichotomized the CRAI variables to include the three highest domain scores versus all other scores (rather than retaining them as continuous exposure variables), we noted the same associations remained significant as in the original analyses. We show these results in Supplementary Table 2. When we examined the domain variables as the difference between the ‘Post’ and ‘Pre’ scores rather than the ‘Post’ scores alone, we observed the same significant associations. We show results for this analysis in Supplementary Table 3. When we examined the continuous outcome variables in their original continuous rather than dichotomous forms, we found significant positive relationships remained between high post-CRAI scores and the course and mentor satisfaction outcome variables. We did not detect significant association for the publication outcome variable. We depict results for this analysis in Supplementary Table 4.

## Discussion

Students completing the SC program at the Johns Hopkins School of Medicine reported significant improvements in their scholarship self-efficacy, with improvements in self-efficacy seen within each domain assessed by the CRAI. In addition, higher final scholarship self-efficacy was significantly associated with satisfaction with the course and mentor, and increased the likelihood of pursuing research in a student’s career. In minimally adjusted models, we observed an association between higher self-efficacy and publications. Our results remained robust to sensitivity analyses, demonstrating that the findings are significant even after accounting for other explanatory factors. This suggests that an SC program is associated with a student’s self-perceived research skills, which may ultimately translate into short- and long-term outcomes useful for medical school programs. Our results are also particularly meaningful because they both track a student’s sense of self-efficacy, and utilize various levels of Kirkpatrick's educational outcome variables, spanning a trajectory of outcome levels from student satisfaction to behavioral change [18]. We, therefore, attempted to be student-centric in both perceptions and behaviors, with a focus on whether students were satisfied with the program and whether it ultimately influenced their career goals.

The most common themes for SC courses are the important value of ownership and strong mentorship in catalyzing and cultivating a passion for future scholarly endeavors. A program similar to the Johns Hopkins SC curriculum was developed at the University of California, San Francisco with the aim of teaching students and trainees the skills needed for a medical education scholarship. Participants specifically identified exposure to and interaction with role models and mentors as a particularly influential component of the experience that inspired them to pursue future career paths in academic medicine [20]. Another similar program was implemented at the Vanderbilt University School of Medicine, with comparable positive course satisfaction results [21]. Other studies highlighted maintaining participant autonomy in choosing research topics alongside the provision of supportive mentorship communities as important features of these programs [22]. Using trainees at a later stage of development, the ‘Primary Care Scholarly Development Program’, developed by educational leaders at Johns Hopkins and Brown University, worked with primary care residents to cultivate interest in scholarship over 1 year. Again, residents cited excellent mentorship as an essential factor in program success, with the ability to choose their own project topic [23]. Our findings take a more student-centric approach, finding that increased self-efficacy in various domains of clinical research is associated with better outcomes, including satisfaction with mentoring. This more student-centric approach, highlighting the importance of building confidence in research skills, is likely to facilitate effective mentoring and make the student’s relationship with faculty more meaningful.

One limitation of the current study is that we conducted it at a single institution with a research-intensive mission and many students wishing to pursue subspecialties and academic careers. Therefore, the results observed in the SC program at this sort of institution might differ from those seen at one with a stronger focus on primary care or that self-selects for students wishing to pursue primarily clinical careers with more limited research and academic goals. In addition, we do not have data on long-term outcomes nor could we completely account for all threats to internal validity, such as selection bias. For instance, other studies found that the number of publications was higher after graduation for students who had competed an SC or similar program [13,24]. Because of the relatively recent establishment of the SC program at Johns Hopkins, data only exist for students recently completing the program, and, as a required course, we do not have data on students who have not completed the SC program. Many students in the dataset are current medical students or residents and thus have had limited opportunities to publish their work or pursue academic careers. Thus, long-term outcomes such as publication data and who remains in academia are not yet available. Finally, we did not examine gender differences in self-efficacy scores. This will be important to include in future studies, considering that 50.5% of all medical students in 2019 were women [25] and that existing literature shows that women’s self-efficacy tends to be lower in research fields and more variable and dependent on perceived societal norms regarding career choices as compared to men with objectively equivalent abilities [26].

To date, our study is among the first to utilize the change in self-efficacy to explore the success of a Scholarly Concentrations curriculum. Self-efficacy for research has been shown to increase between matriculation and graduation of medical school, and self-efficacy at graduation correlated with interest in pursuing future research careers [27]. However, this study by Bierer did not specifically examine changes in self-efficacy in individual learners during an SC program as we have. Future research could expand these quantitative methods to multiple institutions to establish guidelines for designing and evaluating SC programs.

Qualitative data from multiple institutions do exist to provide general guidelines for how successful SC programs are structured. For example, one study identified a focus on developing rigorous critical thinking, analysis, creativity, and synthesis skills alongside the establishment of longitudinal mentorship as hallmarks of a strong SC program [9]. Others suggest a targeted assessment driven by trainee responses to develop evidence-based guidelines for SC programs, such as specific details on the ideal range of topic concentrations to offer [28]. Our current study uses self-efficacy measurement as a tool for applying a student-centered approach to evaluate the SC program, and thus could be used as a starting point for this sort of assessment and guideline development. Finally, recent work has investigated the translation of SC programs to international settings, highlighting specific areas of promise and challenge, and proposing follow-up investigations [15].

## Conclusions

A medical school SC program is one possible way to increase a student’s self-efficacy in conducting scholarship. Higher achieved self-efficacy also may translate to increased satisfaction with the experience and increased likelihood to pursue research. Other medical schools could use such a tool of self-efficacy to both investigate the overall SC experience and understand factors that may increase interest in future physician-scientist pathways.

## Supplementary Material

Supplemental Material
