# Supplementary material for: A medical student scholarly concentrations program: scholarly self-efficacy and impact on future research activities
Source: Med Educ Online. 2020 Jun 26;25(1):1786210. doi: 10.1080/10872981.2020.1786210 (PMC7482758; doi:10.1080/10872981.2020.1786210)
Supplement: Supplemental Material [file ZMEO_A_1786210_SM2147.docx]

**A Medical Student Scholarly Concentrations Program: Scholarly Self-Efficacy and Impact on Future Research Activities**

**Supplementary Material**

**Supplementary Methods: Descriptions of Specific Variables Comprising Mean CRAI Domain Scores**

**Supplementary Table 1: Associations of Average Self-Efficacy Domain Score at End of SC Course with Outcomes, in Sequential Models Results**

**Supplementary Table 2: Sensitivity Analysis of Associations of Average Self-Efficacy Score at End of SC Course with Outcomes, in Sequential Models Results when Highest 3 CRAI Domain Scores Versus All Other Scores Used**

**Supplementary Table 3: Sensitivity Analysis of Associations of Average Self-Efficacy Score at End of SC Course with Outcomes, in Sequential Models Results when Change Variable (Post-Pre CRAI Domain Scores) Used Instead of Post CRAI Variables**

**Supplementary Table 4: Sensitivity Analysis of Associations of Average Self-Efficacy Score at End of SC Course with Outcomes, in Sequential Models When Satisfaction and Publications Outcome Variables Examined as Continuous Rather than Dichotomous**

**Supplementary Methods: Descriptions of Specific Variables Comprising Mean CRAI Domain Scores**

**Study Design & Data Analysis:**

- Choose an appropriate research design that will answer a set of research questions.*
- State the purpose, strengths and limitations of each study design.
- Design a study using quantitative methods, e.g. experimental, quasi-experimental.*^†^
- Determine how each variable will be measured.*
- Select methods of data collection appropriate to the study population and variable.
- Select reliable and valid instruments to measure or assess variables.*
- Design the best data analysis strategy for your study.
- Evaluate the reliability and validity of a given measurement.*
- Analyze data according to their level of measurement and the research design.*
- Perform commonly used statistical tests, such as chi-square, t-tests, analysis of variance, etc.*
- Use computer software to generate graphic images.*
- Explain the outcome of a given analysis in terms of the originally stated hypothesis.
- Express appropriate methodological and theoretical cautions in interpreting results.
- Identify limitations of a study.
- Compare major types of studies (such as case reports, case control, cross-sectional).*^§^
- Recognize important threats to internal and external validity applicable to each.*^§^
- Determine the population and appropriate sample for a given study.*^§^
- Determine an adequate number of subjects for your research project.*^§^

**Conceptualizing a Study:**

- Select a suitable topic area for study.
- Refine a problem so it can be investigated.
- Develop a logical rationale for a particular research idea.
- Organize your proposed research ideas in writing.
- Articulate a clear purpose for the research.
- Place your study in the context of existing research and justify how it contributes.
- Relate specific questions of interest to underlying theory.

**Collaborating with Others:**

- Consult senior researchers for ideas.
- Identify faculty collaborators from within and outside the discipline.
- Initiate research collaborations with colleagues.
- Participate in generating collaborative research ideas.
- Work independently in a research group.
- Adhere to a timeline for research projects.
- Apply a logical approach to designing an educational program.
- Prepare and present a policy brief on a health care issue.
- Apply a logical approach to evaluating an educational program.

**Organizing a Study:**

- Maintain an organized system for ideas and references.
- Maintain a log of your research process (experiments conducted, major decisions, etc.).
- Construct a plan for managing data files.

**Protecting Research Subjects & Responsible Conduct of Research:**

- Describe appropriate recruitment and retention methods used.
- Apply the appropriate process for obtaining informed consent from research subjects.
- Write a human subjects consent form containing the appropriate elements.

**Reporting a Study:**

- Integrate the research findings into the existing literature.
- Select a journal for a manuscript submission.
- Write a literature review that critically synthesizes the relevant literature.
- Write a methods section that conveys sufficient methodological detail to permit.
- Write the results section of a research paper that clearly summarizes and describes the results.
- Write a discussion section for a research paper that articulates its importance.
- Design visual presentations (posters, slides, graphs, pictures).
- Orally present results at a regional or national meeting.
- Defend results to a critical audience.

* Removed from the CRAI-SF survey for the History of Medicine concentration.

† Modified to read “design a study using appropriate methods, e.g. quantitative, qualitative, mixed-methods” for Humanism & Ethics in Medicine concentration.

§ Removed from the CRAI-SF survey for the Basic Science concentration.

**Supplementary Table 1: Associations of Average Self-Efficacy Domain Score at End of SC Course with Outcomes, in Sequential Models**

|  |  | **Model 1** | | **Model 2** | | **Model 3** | |
| --- | --- | --- | --- | --- | --- | --- | --- |
| **Outcome** | **CRAI Domains** | **Odds Ratio** | **95% CI** | **Odds Ratio** | **95% CI** | **Odds Ratio** | **95% CI** |
| **Highest Satisfaction with Course** | Study Design & Data Analysis | **1.38** | **1.13, 1.68** | **1.42** | **1.16, 1.74** | **1.37** | **1.08, 1.73** |
|  | Conceptualizing a Study | **1.36** | **1.09, 1.70** | **1.37** | **1.10, 1.72** | **1.32** | **1.02, 1.72** |
|  | Collaborating with Others | **1.63** | **1.24, 2.13** | **1.73** | **1.28, 2.32** | **1.93** | **1.34, 2.77** |
|  | Organizing a Study | **1.46** | **1.15, 1.85** | **1.49** | **1.16, 1.90** | **1.55** | **1.16, 2.08** |
|  | Protecting Subjects & Responsible Conduct | **1.18** | **1.03, 1.36** | **1.17** | **1.00, 1.35** | 1.13 | 0.95, 1.34 |
|  | Reporting a Study | **1.41** | **1.15, 1.72** | **1.44** | **1.17, 1.77** | **1.42** | **1.11, 1.81** |
| **Highest Satisfaction with Mentor** | Study Design & Data Analysis | **1.37** | **1.15, 1.63** | **1.4** | **1.17, 1.68** | **1.37** | **1.11, 1.70** |
|  | Conceptualizing a Study | **1.34** | **1.10, 1.63** | **1.33** | **1.10, 1.62** | **1.27** | **1.01, 1.61** |
|  | Collaborating with Others | **1.28** | **1.05, 1.55** | **1.3** | **1.06, 1.59** | 1.19 | 0.94, 1.51 |
|  | Organizing a Study | **1.27** | **1.05, 1.53** | **1.27** | **1.05, 1.54** | 1.23 | 0.98, 1.54 |
|  | Protecting Subjects & Responsible Conduct | 1.08 | 0.95, 1.22 | 1.08 | 0.95, 1.24 | 1.05 | 0.90, 1.23 |
|  | Reporting a Study | **1.34** | **1.13, 1.59** | **1.38** | **1.16, 1.65** | **1.45** | **1.16, 1.80** |
| **Published Manuscript During SC** | Study Design & Data Analysis | 1.19 | 0.94, 1.50 | 1.16 | 0.91, 1.48 | 1.07 | 0.80, 1.42 |
|  | Conceptualizing a Study | 1.03 | 0.79, 1.35 | 1.02 | 0.77, 1.35 | 0.892 | 0.62, 1.29 |
|  | Collaborating with Others | 1.17 | 0.95, 1.44 | 1.11 | 0.89, 1.39 | 1.05 | 0.80, 1.38 |
|  | Organizing a Study | 1.17 | 0.96, 1.43 | 1.16 | 0.94, 1.43 | 1.15 | 0.90, 1.48 |
|  | Protecting Subjects & Responsible Conduct | 1.02 | 0.89, 1.18 | 1.02 | 0.88, 1.19 | 1.01 | 0.85, 1.21 |
|  | Reporting a Study | 1.21 | 0.98, 1.50 | 1.2 | 0.96, 1.50 | 1.16 | 0.87, 1.55 |
| **First Author Publication During SC** | Study Design & Data Analysis | 1.19 | 0.97, 1.47 | 1.17 | 0.95, 1.44 | 1.05 | 0.82, 1.33 |
|  | Conceptualizing a Study | 1.23 | 0.97, 1.56 | 1.21 | 0.95, 1.53 | 1.09 | 0.82, 1.43 |
|  | Collaborating with Others | **1.27** | **1.05, 1.53** | **1.23** | **1.01, 1.49** | 1.12 | 0.89, 1.41 |
|  | Organizing a Study | **1.24** | **1.04, 1.49** | **1.22** | **1.02, 1.47** | 1.17 | 0.94, 1.45 |
|  | Protecting Subjects & Responsible Conduct | 1.05 | 0.94, 1.18 | 1.04 | 0.92, 1.18 | 0.981 | 0.85, 1.14 |
|  | Reporting a Study | **1.32** | **1.08, 1.60** | **1.3** | **1.06, 1.59** | 1.19 | 0.93, 1.52 |
| **Likelihood to Conduct Future Research** | Study Design & Data Analysis | 1.13 | 0.96, 1.33 | 1.14 | 0.97, 1.34 | **1.23** | **1.00, 1.50** |
|  | Conceptualizing a Study | **1.26** | **1.04, 1.53** | **1.27** | **1.04, 1.54** | **1.4** | **1.10, 1.78** |
|  | Collaborating with Others | 1.12 | 0.93, 1.35 | 1.16 | 0.95, 1.42 | **1.29** | **1.01, 1.65** |
|  | Organizing a Study | 1.14 | 0.95, 1.36 | 1.14 | 0.94, 1.37 | 1.24 | 0.99, 1.55 |
|  | Protecting Subjects & Responsible Conduct | 1.07 | 0.95, 1.21 | 1.04 | 0.92, 1.18 | 1.09 | 0.94, 1.26 |
|  | Reporting a Study | **1.19** | **1.01, 1.41** | **1.19** | **1.01, 1.41** | **1.37** | **1.10, 1.69** |

Abbreviations: CRAI = Clinical Research Appraisal Inventory, using the Post-SC questionnaire as measures of self-efficacy attainment; 95% CI = 95% Confidence Intervals.

Average CRAI Score obtained by averaging the scores on questions in each domain of the CRAI.

Model 1: Unadjusted Model, containing only exposure and outcome variables.

Model 2: Minimally Adjusted Model, containing exposure and outcome variables, as well as SC concentration category.

Model 3: Fully Adjusted Model, containing exposure and outcome variables, covariates from Model 2, as well as publications prior to SC and average CRAI domain score before starting the SC curriculum.

**Supplementary Table 2: Sensitivity Analysis of Associations of Average Self-Efficacy Score at End of SC Course with Outcomes, in Sequential Models Results when Highest 3 CRAI Domain Scores Versus All Other Scores Used**

| **Outcome** | **CRAI Domains** | **Odds Ratio** | **95% CI** |
| --- | --- | --- | --- |
| **Highest Satisfaction with Course** | Average CRAI Post Value | **1.57** | **1.20, 2.07** |
| **Highest Satisfaction with Mentor** | Average CRAI Post Value | **1.46** | **1.15, 1.86** |
| **Published Manuscript During SC** | Average CRAI Post Value | 1.09 | 0.79, 1.52 |
| **First Author Publication During SC** | Average CRAI Post Value | 1.13 | 0.85, 1.48 |
| **Likelihood to Conduct Future Research** | Average CRAI Post Value | **1.46** | **1.15, 1.86** |

Abbreviations: CRAI = Clinical Research Appraisal Inventory, using mean domain scores on the Post-SC questionnaire as measures of self-efficacy attainment; 95% CI = 95% Confidence Intervals.

Average CRAI Score obtained by averaging the scores on questions in each domain of the CRAI, and then averaging across the 6 domains. Results presented include the Fully Adjusted Model, containing exposure and outcome variables, choice of SC concentration, as well as publications prior to SC and average CRAI score before starting the SC curriculum.

**Supplementary Table 3: Sensitivity Analysis of Associations of Average Self-Efficacy Score at End of SC Course with Outcomes, in Sequential Models Results when Change Variable (Post-Pre CRAI Domain Scores) Used Instead of Post CRAI Variables**

| **Outcome** | **CRAI Domains** | **Odds Ratio** | **95% CI** |
| --- | --- | --- | --- |
| **Highest Satisfaction with Course** | Average CRAI Post Value | **1.50** | **1.15, 1.96** |
| **Highest Satisfaction with Mentor** | Average CRAI Post Value | **1.40** | **1.11, 1.77** |
| **Published Manuscript During SC** | Average CRAI Post Value | 1.10 | 0.79, 1.52 |
| **First Author Publication During SC** | Average CRAI Post Value | 1.12 | 0.85, 1.48 |
| **Likelihood to Conduct Future Research** | Average CRAI Post Value | **1.51** | **1.19, 1.92** |

Abbreviations: CRAI = Clinical Research Appraisal Inventory, using mean domain scores on the Post-SC questionnaire as measures of self-efficacy attainment; Regression Coefficient = Beta coefficient generated with linear regression models; 95% CI = 95% Confidence Intervals.

Average CRAI Score obtained by averaging the scores on questions in each domain of the CRAI, and then averaging across the 6 domains. Results presented include the Fully Adjusted Model, containing exposure and outcome variables, choice of SC concentration, as well as publications prior to SC and average CRAI score before starting the SC curriculum.

**Supplementary Table 4: Sensitivity Analysis of Associations of Average Self-Efficacy Score at End of SC Course with Outcomes, in Sequential Models When Satisfaction and Publications Outcome Variables Examined as Continuous Rather than Dichotomous**

| **Outcome** | **CRAI Domains** | **Regression Coefficient** | **95% CI** |
| --- | --- | --- | --- |
| **Highest Satisfaction with Course** | Average CRAI Post Value | **0.22** | **0.13, 0.31** |
| **Highest Satisfaction with Mentor** | Average CRAI Post Value | **0.16** | **0.08, 0.24** |
| **Published Manuscript During SC** | Average CRAI Post Value | 0.36 | -0.16, 0.87 |

Abbreviations: CRAI = Clinical Research Appraisal Inventory, using mean domain scores on the Post-SC questionnaire as measures of self-efficacy attainment; Regression Coefficient = Beta coefficient generated with linear regression models; 95% CI = 95% Confidence Intervals.

Average CRAI Score obtained by averaging the scores on questions in each domain of the CRAI, and then averaging across the 6 domains. Results presented include the Fully Adjusted Model, containing exposure and outcome variables, choice of SC concentration, as well as publications prior to SC and average CRAI score before starting the SC curriculum.
